# Comparison of concurrent chemoradiotherapy versus neoadjuvant chemotherapy followed by radiation in patients with advanced nasopharyngeal carcinoma in endemic area: experience of 128 consecutive cases with 5 year follow-up

**DOI:** 10.1186/1471-2407-14-787

**Published:** 2014-10-28

**Authors:** Shang-Yin Wu, Yuan-Hua Wu, Ming-Wei Yang, Wei-Ting Hsueh, Jenn-Ren Hsiao, Sen-Tien Tsai, Kwang-Yu Chang, Jeffrey S Chang, Chia-Jui Yen

**Affiliations:** Division of Hematology/Oncology, Department of Internal Medicine, National Cheng Kung University Hospital, College of Medicine, National Cheng Kung University, 138 Sheng-Li Road, Tainan, 704 Taiwan; Department of Radiation Oncology, National Cheng Kung University Hospital, College of Medicine, National Cheng Kung University, Tainan, Taiwan; Department of Otorhinolaryngology, National Cheng Kung University Hospital, College of Medicine, National Cheng Kung University, Tainan, Taiwan; National Institute of Cancer Research, National Health Research Institutes, Tainan, Taiwan

**Keywords:** Neoadjuvant chemotherapy, Concurrent chemoradiation, Nasopharyngeal carcinoma, Outcome

## Abstract

**Background:**

Combined radiotherapy and chemotherapy is considered the standard of care for locally advanced nasopharyngeal carcinoma (LA-NPC) in Epstein-Barr virus infection endemic area. This study compared the long-term outcomes between LA-NPC patients treated with neoadjuvant chemotherapy followed by radiotherapy (NACT) and those treated with concurrent chemoradiotherapy (CCRT).

**Methods:**

From 2003 to 2007, a total of 128 histopathologically proven LA-NPC patients receiving either NACT or CCRT were consecutively enrolled at the National Cheng Kung University Hospital in Taiwan. NACT consisted of 3-week cycles of mitomycin, epirubicin, and cisplatin on day 1 and fluorouracil and leucovorin on day 8 (MEPFL) or weekly alternated cisplatin on day 1 and fluorouracil and leucovorin on day 8 (P-FL). CCRT comprised 3-week cycles of cisplatin (Cis 100) or 4-week cycles of cisplatin and fluorouracil (PF4). The first failure site, disease free survival (DFS), overall survival (OS), and other prognostic factors were analyzed.

**Results:**

Thirty-eight patients (30%) received NACT. Median follow-up duration was 53 months. More patients with advanced nodal disease (N2-N3) (86.8% vs 67.8%, *p* =0.029) and advanced clinical stage (stage IVA-IVB) enrolled in the NACT group (55.2% vs 26.7%, *p* =0.002). For NACT, both MEPFL and P-FL had similar 5-year DFS and OS (52.9% vs 50%, *p* =0.860 and 73.5% vs 62.5%, *p* =0.342, respectively). For CCRT, both PF4 and Cis 100 had similar 5-year DFS and OS (62.8% vs 69.6%, *p* =0.49 and 72.9% vs 73.9%, *p* =0.72, respectively). Compared to CCRT, NACT had similar 5-year DFS and OS (51.5% vs 65.1%, *p* =0.28 and 71.7% vs 74.3%, *p* =0.91, respectively). Among patients who were recurrence-free in the first 2 years after treatment, those treated with NACT experienced poorer locoregional control compared to those treated with CCRT (Hazard ratio =2.57, 95% confidence interval: 1.02 to 6.47, *p* =0.046).

**Conclusions:**

For LA-NPC, both CCRT and NACT were similarly efficacious treatment strategies in terms of long-term disease control and survival probability. Close locoregional follow-up is recommended for patients receiving NACT, because these patients are more prone to develop locoregional failure than patients receiving CCRT.

## Background

Nasopharyngeal carcinoma (NPC) is an Epstein-Barr virus-associated cancer with a high incidence in Southeast Asia, including Taiwan. The majority of NPC cases in Southeast Asia are classified as WHO undifferentiated type III [1], which differs from the WHO type I NPC commonly seen in the Western counties and is relatively sensitive to radiotherapy (RT) and chemotherapy [2, 3]. For early stage disease, RT is the mainstay treatment modality with a 5-year overall survival of 75-90% [1, 4]. For locally advanced NPC, combined chemotherapy with RT may prolong overall survival with an absolute 5-year survival benefit of 4% [5–7].

In a recent meta-analysis, Langendijk *et al*. showed that the most efficacious way to introduce chemotherapy was concurrently with radiotherapy and this approach resulted in an absolute 5-year survival benefit of 20% [5]. But Zhang *et al*. reported that the relative benefit of this approach might be less in NPC-endemic area than that in non-NPC-endemic area [8] and Lee *et al*. reported after median follow-up 5.9 years, the administration of cisplatin plus adjuvant cisplatin-fluorouracil concurrent with radiotherapy showed a better 5-year progression free survival but also greater incidence of acute toxicity and no 5-year overall survival difference compared with radiotherapy alone [9]. In addition, the pivotal INT-0099 trial showed that 37% of patients in the concurrent chemoradiotherapy arm prematurely terminated the treatment because of excess toxicity [10].

Neoadjuvant (induction) chemotherapy before radiotherapy may significantly reduce the risk of locoregional recurrence and distant metastases and may improve disease specific survival in locally advanced NPC [11]. This approach may eradicate micrometastases, facilitate the planning of radiotherapy, and improve local disease control by reducing the tumor volume prior to RT [12]. Two studies conducted in NPC-endemic area by Hong *et al*. [13] and Lin *et al*. [14] reported that after neoadjuvant chemotherapy over 90% of NPC patients were able to complete definitive radiotherapy, which is considered the mainstay treatment in NPC. Therefore, for patients with locally advanced NPC in NPC-endemic area, the best timing to incorporate chemotherapy with radiotherapy is still an unresolved issue [8]. Despite the proven advantage of neoadjuvant or concurrent chemotherapy with radiotherapy over the radiation alone, the direct comparison of these two treatment strategies in a study with large sample size (>100 patients) and long-term (>5 years) follow-up has not been conducted.

The aim of present study was to evaluate the long-term outcome difference between neoadjuvant chemotherapy followed by radiotherapy (NACT) and concurrent chemoradiotherapy (CCRT) in patients with locally advanced NPC in NPC-endemic area.

## Methods

Between 2003 and 2007, patients with pathologically confirmed, previously untreated stage IIB to stage IVB NPC according to the 2002 American Joint Committee on Staging of Cancer classification [15], who received study-defined neoadjuvant chemotherapy as well as who received concurrent chemoradiotherapy in the National Cheng Kung University Hospital (NCKUH) in Tainan, Taiwan were consecutively enrolled. Treatment decisions were made by an institutional tumor board consisted of otolaryngologists, medical oncologists and radiation oncologists. Either NACT or CCRT was an accepted treatment option during that period.

Pretreatment evaluation for eligible subjects included a general physical examination, fiberoptic endoscopy, contrast-enhanced computed tomography (CT) and/or magnetic resonance imaging (MRI) of the nasopharynx and neck, chest x-ray, hepatic ultrasonography, and radionuclide bone scan.

In general, we followed the criteria reported by Lin *et al*. [14] to select patient to receive NACT: (1) neck nodal size >6 cm; (2) supraclavicular node metastasis; (3) skull base destruction/intracranial invasion, and (4) multiple neck nodes metastasis with one of nodal size >4 cm. In addition, if a patient could not accept a delay in radiation simulation, we also assigned him/her to receive NACT.

To be eligible, all patients had to receive study-defined chemotherapy regimen with no distant metastasis and no concurrent malignancies. Patients who had no subsequent clinical follow-up data, did not receive tumor board-defined treatment options or died during chemotherapy and/or radiotherapy treatment period were excluded from the current study.

The study was approved by the Institutional Review Board of National Cheng Kung University Hospital and preceded according to the Helsinki Declaration.

### Treatment

For patients receiving NACT, the study-defined neoadjuvant chemotherapy consisted of MEPFL regimen (mitomycin 8 mg/m^2^, epirubicin 60 mg/m^2^, and cisplatin 60 mg/m^2^ on day 1 and 5-fluorouracil [5-FU] 450 mg/m^2^ plus leucovorin 30 mg/m^2^ on day 8, 3-week cycle for 3 cycles) or weekly P-FL regimen (cisplatin 60 mg/m^2^ 3-h infusion alternating with 5-FU 2500 mg/m^2^ plus leucovorin 250 mg/m^2^ continuous infusion for 24 h weekly for a total of 10 weeks) then followed by radiotherapy alone as defined by previous studies [13, 14].

For patients receiving CCRT, we followed the protocol of Lin *et al*. (cisplatin 20 mg/m^2^/day plus fluorouracil 400 mg/m^2^/day by 96-hour continuous infusion during weeks 1 and 5 of radiotherapy; PF4 regimen) or AI-Sarraf M *et al*. (cisplatin 100 mg/m^2^ on day 1, 22, and 43 during radiotherapy; Cis 100 regimen) [10, 16].

Chemotherapy modification was permitted at the discretion of the primary treating physician when patients experienced grade 3 toxicity and the modification was guided by the designs of the original trials.

### Radiotherapy

Intensity Modulated Radiotherapy (IMRT; Clinac iX accelerator, Varian) was equipped in our hospital in 2006. All patients treated after May 2006 used IMRT technique except one patient who received radiosurgery boost.

In the 2D method, we used bilaterally opposed fields with a matched lower anterior neck field first. The bilateral opposing fields were then coned down to the high-risk regions that included nasopharynx after 43.2 Gy to avoid spinal cord damage. The posterior neck node regions were boosted with 9-12 MeV electron beam. Nasopharyngeal tumor and gross nodes were boosted by 3D conformal RT or a frameless stereotactic body radiation therapy (SBRT; Cyberknife) to the planned dose [17].

Among patients receiving IMRT, the inverse planning software (Eclipse treatment planning system, Varian) was used. The high-risk clinical target volume (CTV) covered the entire nasopharynx, tumor invasion areas, and gross nodes. The intermediate-CTV included the suspected nodes plus the involving neck levels. The low-risk CTV included other lower risk lymphatic regions for occult micrometastases. The low neck lymphatic regions were treated with the same IMRT plan. Planning target volumes (PTVs) were created by automated expansion of 3 to 5 mm of all CTVs to account for setup error. Normal structures, including the parotid glands, spinal cord, brain stem, optic nerves, and optic chiasm were also contoured on the treatment plan.

### Assessment and follow-up

All patients had regular follow-up with radiation oncologists at the outpatient clinic. Patients received regular clinical examinations every 1–3 months in the first year after the completion of therapy, every 3 months during the second and third years, and then every 4–6 months thereafter. Regular follow-up MRI was performed every 3-6 months after the completion of radiation therapy and then every 6-12 months thereafter if no gross tumor recurrence was noted clinically.

### Statistical analysis

We collected the patients’ demographic data, including gender and age, histology type, and stage as well as chemotherapy regimen and given cycles, radiotherapy dose and elapsed days. The location of the first clinical relapse was recorded and classified as locoregional failure if the first relapse site was nasopharynx or neck nodal area and as distant failure if relapse site was beyond the above-mentioned areas. The duration of disease-free survival (DFS), time to locoregional failure (TTLF) and time to distant failure (TTDF) were calculated from Day 1 of the treatment until documented treatment failure, death from any cause or the date of last follow-up, whichever came first. The duration of overall survival (OS) was measured from Day 1 of the treatment until death or the date of last follow-up.

Statistical analyses were performed using SPSS statistical program (SPSS for Windows, release 17.0; SPSS, Chicago, IL, USA) and SAS 9.2 (Cary, NC, USA). The Pearson χ^2^ test or Fisher’s exact test (if the expected number was less than five in at least one cell) for comparison of categorical variables and independent *t* test for comparison of continuous variables were applied. To evaluate the survival of the patients, we used the Kaplan-Meier test to construct survival curves, which were compared using log-rank tests. Kaplan-Meier survival curves were drawn using GraphPad Prism 4 (La Jolla, CA, USA). Hazard ratio (HR) and the associated 95% confidence interval (CI) were calculated using Cox regression model. For all statistical analyses, a two-sided *P* value <0.05 was considered statistically significant.

## Results

### Patient characteristics

From 2003 to 2007, a total of 128 eligible patients were consecutively enrolled. The median age was 48 years (range: 16-81 years). Thirty-eight patients (30%) received NACT and 90 patients received CCRT. Patients’ disease characteristics and demographics were well-balanced in each group except more patients with advanced nodal disease (N2-N3) (86.8% vs 67.8%, *p* =0.029) and advanced clinical stage (stage IVA-IVB) enrolled in the NACT group (55.2% vs 26.7%, *p* =0.002) (Table 1).

**Table 1:** **Patient demographics and disease characteristics**

	CCRT group (n = 90)		NACT group (n = 38)		***P***-value
Characteristics	No.	%	No.	%	
Age, years					0.95
Range	15.7 - 81.5		22.9 - 73.0		
Median	48.2		48.2		
Mean	47.9		47.8		
Gender					0.347
Male	73	81.1	28	73.7	
Female	17	18.9	10	26.3	
WHO Classification					0.941
Type I	4	4.4	2	5.3	
Type II	31	34.4	12	31.6	
Type III	55	61.1	24	63.2	
T stage					0.200
T1 to T2	56	62.2	19	50	
T3 to T4	34	37.8	19	50	
N stage					0.029
N0 to N1	29	32.2	5	13.2	
N2 to N3	61	67.8	33	86.8	
Overall Stage					0.002
IIB-III	66	73.3	17	44.8	
IVA-IVB	24	26.7	21	55.2	

CCRT, concurrent chemoradiotherapy; NACT, neoadjuvant chemotherapy followed by radiotherapy; WHO, World Health Organization.

### Treatment administration and compliance

In the NACT group, six of eight patients (75%) completed the planned three-cycle MEPFL and twenty-nine of thirty patients (96.7%) finished the ten-week P-FL neoadjuvant chemotherapy, which were comparable to those reported by previous studies (90% for MEPFL reported by Hong *et al*. [13] and 93% for P-FL reported by Lin *et al*. [14]).

In the CCRT group, sixty four of sixty-six (97%) and twenty of twenty-four patients (83.3%) received concurrent chemoradiotherapy with PF4 for two times and Cis 100 every three weeks, respectively, which were similar to those previously reported (93.6% for PF4 reported by Lin *et al*. [16] and 62.8% for Cis 100 reported by AI-Sarraf M *el al*. [10]).

A total of 61 patients received the traditional 2D radiotherapy followed by conformal RT or radiosurgery boost, while 67 patients received a full course of IMRT. As for the radiation compliance, the median RT dose and the median duration of RT were no significantly different between the NACT group and the CCRT group (*p* =0.123 and 0.772 respectively) (Table 2) and none of the patients died within 90 days after radiotherapy completed.

**Table 2:** **Compliance with radiotherapy**

	CCRT group (n = 90)	NACT group (n = 38)	***P***-value
RT Technique			0.667
2D, n (%)	44 (48.9)	17 (44.7)	
IMRT, n (%)	46 (51.1)	21 (55.3)	
RT Compliance			
Median dose RT, cGy (range)	7380 (6920 - 7860)	7200 (6840 - 7720)	0.123
Median duration of RT, Days (range)	58 (51 - 84)	57 (48 - 81)	0.772

### Efficacy

After a median follow-up of 53 months (range: 7 to 101 months), twenty-one (16.4%) and twenty-two (17.2%) patients developed locoregional and distant failure as the first failure site, respectively. And for non-recurrent subjects, the median follow-up time was 59 months (range: 41 to 101 months). The frequency, site of first recurrence and the first salvage treatment modality for locoregional recurrence in each treatment group are summarized in Table 3. Notably, compared with the CCRT group, the locoregional control appeared poorer for the NACT group (23.7% vs 13.3%).

**Table 3:** **Frequency, site of first recurrence and first salvage treatment modality for locoregional recurrence**

	CCRT group (n = 90)		NACT group (n = 38)	
	No.	%	No.	%
First failure site				
Locoregional	12	13.3	9	23.7
Distant	14^a^	15.6	8	21.1
Lung	4		4	
Bone	6		2	
Liver	5		1	
Others	2^b^		1^c^	
First treatment modality for locoregional failure				
Surgery	1	8.3	-	
Re-irradiation	1	8.3	6	66.7
Re-CCRT	5	41.7	3	33.3
Chemotherapy	5	41.7	-	

^a^One patient had bone and lung metastases concurrently and the other one had bone, liver and lung metastases simultaneously ^b^one patient developed mediastinal lymph node metastases and the other one developed para-aortic lymph node metastases ^c^the subject developed axillary lymph node metastases.

For the 38 patients in the NACT group, the 3-year and 5-year DFS rate were 60.5% and 51.5%, respectively, and the 3-year and 5-year OS rate were 86.8% and 71.7% respectively. Compared with patients who received MEPFL regimen, patients who received P-FL regimen had similar 5-year DFS and OS (52.9% vs 50%, *p* =0.860 and 73.5% vs 62.5%, *p* =0.342, respectively).

For the 90 patients in the CCRT group, the 3-year and 5-year DFS rate were 72.2% and 65.1%, respectively, and the 3-year and 5-year OS rate were 83.3% and 74.3%, respectively. Compared with patients who received Cis 100 regimen, patients who received PF4 regimen had similar 5-year DFS and OS without statistical difference (62.8% vs 69.6%, *p* =0.49 and 72.9% vs 73.9%, *p* =0.72, respectively).

The 5-year DFS rate in the NACT group and the CCRT group was 51.5% and 65.1% respectively (*p* =0.28). Advanced clinical T stage (T3-4) and advanced clinical overall stage (stage IVA-IVB) were associated with a shorter disease free survival (HR =2.19, 95% CI: 1.24 to 3.85, *p* =0.007 and HR =2.7, 95% CI: 1.54 to 4.74, *p* =0.0006, respectively). The DFS was not influenced by other clinical parameters, including gender (*p* =0.33), WHO histology classification (*p* =0.72), or nodal status (*p* =0.35) (Table 4). NPC with advanced clinical stage (stage IVA-IVB) had poor locoregional control (HR = 2.84, 95% CI: 1.19 to 6.77, *p* =0.02) and those receiving NACT tended to have poor locoregional control (HR = 1.75, 95% CI: 0.74 to 4.16, *p* =0.20). Advanced clinical stage (stage IVA-IVB) was the only clinical parameter predicting poor distant control (HR = 2.62, 95% CI: 1.13 to 6.08, *p* =0.02) (Table 5).

**Table 4:** **Univariate Cox regression analysis for disease free survival and overall survival**

	DFS				OS			
	Event	HR	95% CI	***P***-value	Event	HR	95% CI	***P***-value
Gender								
Male (n = 101)	41	1.00			33	1.00		
Female (n = 27)	8	0.69	0.32-1.47	0.33	3	0.35	0.11-1.14	0.08
WHO Classification								
Type I (n = 6)	3	1.00			1	1.00		
Type II (n = 43)	17	0.63	0.18-2.14	0.46	12	1.59	0.21-12.22	0.66
Type III (n = 79)	29	0.61	0.19-2.02	0.42	23	1.68	0.23-12.47	0.61
cT								
T1-2 (n = 75)	21	1.00			13	1.00		
T3-4 (n = 53)	28	2.19	1.24-3.85	0.007	23	2.9	1.47-5.73	0.002
cN								
N0-1 (n = 34)	11	1.00			10	1.00		
N2-3 (n = 94)	38	1.38	0.70-2.70	0.35	26	1.06	0.49-2.13	0.95
Stage								
IIB/III (n = 83)	23	1.00			15	1.00		
IVA/IVB (n = 45)	26	2.70	1.54-4.74	0.0006	21	3.00	1.54-5.83	0.001
Treatment								
CCRT group (n = 90)	31	1.00			25	1.00		
NACT group (n = 38)	18	1.38	0.77-2.46	0.28	11	1.04	0.51-2.12	0.91

DFS, disease free survival; OS, overall survival; HR, hazard ratio; CI, confidence interval.

**Table 5:** **Univariate Cox regression analysis for locoregional failure and distant failure**

	Locoregional failure				Distant failure			
	Event	HR	95% CI	***P***-value	Event	HR	95% CI	***P***-value
Gender								
Male (n = 101)	16	1.00			20	1.00		
Female (n = 27)	5	1.11	0.41-3.04	0.83	2	0.36	0.08-1.53	0.16
WHO Classification								
Type I (n = 6)	1	1.00			1	1.00		
Type II (n = 43)	8	0.98	0.12-7.82	0.98	6	0.75	0.09-6.21	0.79
Type III (n = 79)	12	0.83	0.11-6.39	0.86	15	1.05	0.14-7.94	0.96
cT								
T1-2 (n = 75)	9	1.00			10	1.00		
T3-4 (n = 53)	12	2.11	0.89-5.02	0.09	12	1.90	0.82-4.39	0.14
cN								
N0-1 (n = 34)	5	1.00			3	1.00		
N2-3 (n = 94)	16	1.20	0.44-3.29	0.72	19	2.43	0.72-8.24	0.15
Stage								
IIB/III (n = 83)	9	1.00			10	1.00		
IVA/IVB (n = 45)	12	2.84	1.19-6.77	0.02	12	2.62	1.13-6.08	0.02
Treatment								
CCRT group (n = 90)	12	1.00			14	1.00		
NACT group (n = 38)	9	1.75	0.74-4.16	0.20	8	1.29	0.54-3.08	0.56

**Figure 1:**
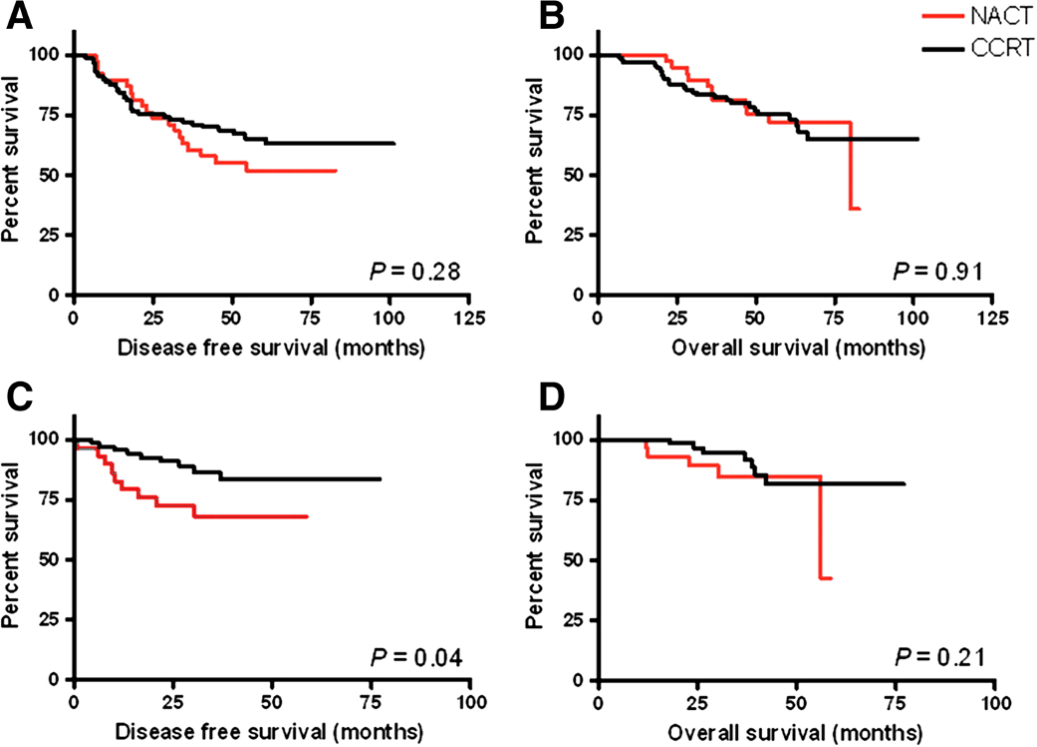
**Kaplan-Meier curves of disease free survival and overall survival.** Kaplan-Meier curves of disease free survival **(A)** and overall survival **(B)** for patients in NACT group (red) in comparison with CCRT group (black). Patients without recurrence or death in first 2 years were further analyzed and disease free survival **(C)** and overall survival **(D)** curve as illustrated.

Notably, the Kaplan-Meier DFS curves of the NACT group and the CCRT group started to separate at 2 years after treatment (Figure 1A). According to past experience, most NPC patients suffered from disease recurrence in the first 2-3 years while completing radical therapy for advanced disease control [13, 18]. When we only considered patients who remained disease-free in the first 2 years for further analysis, compared to those who received CCRT, patients who received NACT had a higher risk for recurrence (HR = 2.57, 95% CI:1.02 to 6.47, *p* =0.046) (Figure 1C). No other clinical parameters, such as gender (*p* =0.26), histology classification (*p* =0.23), clinical T stage (*p* =0.07), clinical nodal stage (*p* =0.44) and clinical stage (*p* =0.49), had a significant influence on late disease recurrence.

**Figure 2:**
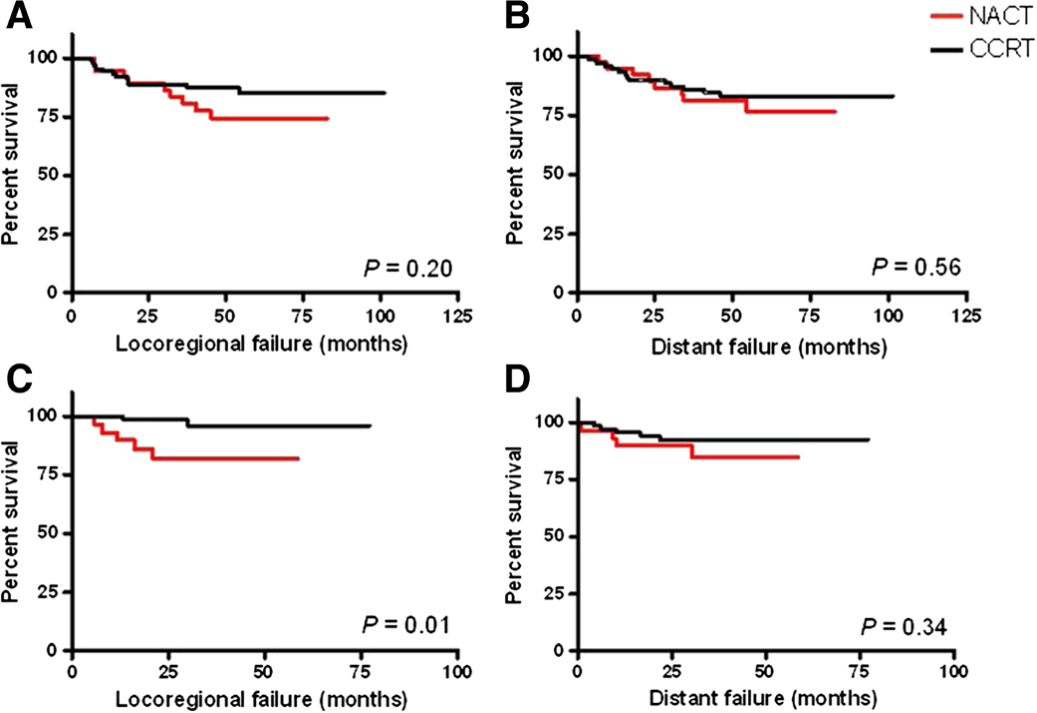
**Kaplan-Meier curves of time to locoregional failure and time to distant failure.** Kaplan-Meier curves of time to locoregional failure **(A)** and time to distant failure **(B)** for patients in NACT group (red) in comparison with CCRT group (black). Patients without recurrence or death in first 2 years were further analyzed and time to locoregional failure **(C)** and time to distant failure **(D)** curve as illustrated.

The 5-year TTLF rate was 85.2% for CCRT and 74.5% for NACT (*p* =0.20) and the 5-year TTDF rate was 83.1% for CCRT and 76.4% for NACT (*p* =0.56). The Kaplan-Meier curve did not separate till 2 years in TTLF analysis (Figure 2A) but not in TTDF analysis (Figure 2B). When we re-analyzed patients who remained disease-free in first 2 years, patients who received NACT had a higher risk for developing locoregional failure (HR =6.31, 95% CI: 1.22 to 32.59, *p* =0.03) but not distant failure (HR =1.87, 95% CI: 0.50 to 6.96, *p* =0.35). No other clinical parameters, such as gender, histology classification, clinical T stage, clinical nodal stage and clinical stage had a significant influence on late locoregional failure or distant failure (Table 6).

**Table 6:** **Univariate Cox regression analysis for locoregional failure and distant failure for subjects without recurrence or death in the first two years**

	Locoregional failure				Distant failure			
	Event	HR	95% CI	***P***Value	Event	HR	95% CI	***P***Value
Gender								
Male (n = 76)	5	1.00			9	1.00		
Female (n = 21)	2	1.41	0.27-7.29	0.68	0	–	–	–
WHO Classification								
Type III (n = 59)	2	1.00			6	1.00		
Type II (n = 35)	5	4.39	0.85-22.62	0.08	3	0.83	0.21-3.33	0.79
Type I (n = 3)	0	–	–	–	0	–	–	–
cT								
T1-2 (n = 62)	3	1.00			4	1.00		
T3-4 (n = 35)	4	2.47	0.55-11.05	0.24	5	2.25	0.61-8.41	0.22
cN								
N0-1 (n = 27)	1	1.00			2	1.00		
N2-3 (n = 70)	6	2.64	0.32-22.02	0.37	7	1.49	0.31-7.19	0.62
Stage								
IIB/III (n = 72)	4	1.00			7	1.00		
IVA/IVB (n = 25)	3	2.31	0.52-10.35	0.27	2	0.79	0.16-3.82	0.77
Treatment								
CCRT group (n = 68)	2	1.00			5	1.00		
NACT group (n = 29)	5	6.31	1.22-32.59	0.03	4	1.87	0.50-6.96	0.35

Thirty-six of 128 patients (28.1%) died during the study period which consisted of 25 in the CCRT group and 11 in the NACT group. The 5-year OS rate in NACT group and CCRT group was 71.7% and 74.3%, respectively (*p* =0.91). Similar to DFS, only advanced T stage (HR = 2.9, 95% CI: 1.47-5.73, *P* =0.002) and advanced clinical stage (HR = 3.0, 95% CI: 1.54-5.83, *P* =0.001) had poor overall survival (Table 4).

Although the NACT group tended to have poorer locoregional control than the CCRT group, more patients survived after salvage therapy in the NACT group (five patients survived among the nine locoregional failure patients) than in the CCRT group (three patients survived among the twelve locoregional failure patients) (Table 3).

## Discussion

For locally advanced NPC, the present study demonstrated that both NACT and CCRT resulted in similar outcomes in terms of 5-year DFS and OS, despite that more patients with advanced nodal disease (N2-N3, 86.8% vs 67.8%, *p* =0.029) and advanced overall clinical stage (stage IVA-IVB, 55.2% vs 26.7%, *p* =0.002) enrolled in NACT group. In addition, our study also showed that for patients who were disease-free in the first two years after the completion of treatment, those who received NACT experienced poorer late locoregional control (HR =2.57, 95% CI: 1.02 to 6.47, *p* =0.046) (Figure 1C). Despite that NACT tended to have poorer locoregional control than CCRT, more patients survived after salvage therapy in the NACT group (five patients survived among the 9 locoregional failure patients) than in CCRT group (three patients survived among the 12 locoregional failure patients), which resulted in a similar long-term OS in both treatment groups.

CCRT is considered the standard of care for locally advanced NPC but the relative benefit of this approach may be less in Epstein-Barr virus infection endemic area than non-endemic area [8, 19]. NACT showed promising result in terms of overall survival and treatment tolerability in the NPC-endemic area and this treatment strategy seems to be another reasonable option at least in the NPC-endemic area [13, 14, 20]. For locally advanced NPC, only limited information regarding the comparison between NACT and CCRT in terms of DFS and OS has been reported and no information on long-term DFS and OS is available.

To the best of our knowledge, only Xu *et al*. and Komatsu *et al*. have ever addressed this issue [21, 22]. In a preliminary report, Xu and colleagues reported a relatively short-term 3-year follow-up result of a phase III randomized study comparing NACT and CCRT for locally advanced NPC [22]. After a median follow-up of 38 months, the 3-year OS was 95.9% vs. 94.5% (*p* =0.54) in NACT and CCRT, respectively, which is better than our 3-year OS result of 83.3% vs. 86.8% (*p* =0.91). This may be partly due to the fact that 45 of 128 (35%) in our cohort were stage IVA-IVB disease, in contrast to the 85 of 338 (25%) in the study by Xu *et al*. In addition, 67% of the study population in Xu *et al*. trial received the planned adjuvant chemotherapy, while none of our study patients received the planned adjuvant chemotherapy. In the DFS analysis by Xu *et al*., the 3-year DFS in the NACT group was inferior than that in the CCRT group (78.5% vs. 82.5%, *p* =0.16) and the Kaplan-Meier curves of the NACT group and the CCRT group separated after 16 months, which was consistent with our finding. Because most NPC patients suffered from disease recurrence during the first 2-3 years while completing radical therapy for advanced disease control [13, 18], we further analyzed patients who remained disease-free in the first two years after the completion of treatment, and found that patients who received NACT experienced poorer late locoregional control (HR =2.57, 95% CI: 1.02 to 6.47, *p* =0.046) compared to those who received CCRT (Figure 1C).

Komatsu *et al*. reported a relatively small series (N =46) of advanced NPC patients receiving either CCRT or NACT with a long-term follow-up (median 51.7 months) [21]. They reported that the 5-year disease specific survival in NACT and CCRT was 67.3% and 60.1%, respectively, which is similar to our finding (in our study, the 5-year rate was 71.7% vs. 74.3%). In addition, the authors also noted that the CCRT group showed less locoregional failure than in the NACT group (12% vs. 19%) and suggested CCRT could provide better locoregional control. Our result is consistent with that reported by Komatsu *et al*. (Figures 1C and 2C) and we further demonstrated that among patients who remained disease-free in the first two years after completion of treatment, those who received NACT experienced poorer late locoregional control than those who received CCRT (Table 6).

Although this is the first observational study to address NACT and CCRT efficacy for locally advanced NPC with a relatively large number of patients (N =128) and a long-term follow-up (median follow-up =53 months) in a NPC-endemic area, several limitations need to be mentioned. First, the study design was retrospective and in order to minimize selection bias, we included all consecutively-enrolled eligible patients in our institute during the study period for the current analysis. Second, we adopted two different chemotherapy regimens in both the NACT and the CCRT group. In the NACT group, we used P-FL or MEP-FL as the NACT regimens and they had a similar drug administration rate, compliance rate, DFS, and OS with each other and with those reported by previous studies. Thus, we combined these two different subgroups together as NACT for further analysis and we did the same thing for the CCRT group. Third, we reported the long-term follow-up outcome without reporting the post-treatment response because the aim of this study was to determine the long-term efficacy of NACT and CCRT and not the treatment response. Besides, the slowing regressing tumor or a truly residual tumor in post-treatment locally advanced NPC patients may be difficult to differentiate without surgical confirmation [1, 23] and not all of the suspicious patients in our cohort received post-treatment nasopharyngeal biopsy to have a complete report of the treatment response. Finally, we did not report treatment-related toxicities in this retrospective study because the physicians only recorded the most significant adverse effect in daily practice instead of recording all adverse effects as required in a clinical trial and this could have resulted in the underestimation of the minor significant adverse effects.

## Conclusions

In conclusion, our study demonstrated that both CCRT and NACT are similarly efficacious treatment strategies in terms of long-term DFS and OS for locally advanced NPC in Taiwan and possibly in the other Epstein-Barr viral infection endemic areas. For patients receiving NACT, a long-term follow-up is recommended because these patients are more prone to develop locoregional failure than patients receiving CCRT. It seems that neoadjuvant chemotherapy followed by concurrent chemoradiation therapy can provide the best survival benefit for LA-NPC but several studies have generated conflicting results and this issue needs further investigation with a proper study design and patient selection [20, 24, 25]. The long-term follow-up result from the phase III study of Xu *et al*. [22] will provide more direct evidence and knowledge about the role of NACT for LA-NPC in NPC-endemic area.
